# Genetic alterations in chronic lymphocytic leukemia and plasma cell neoplasms – a practical guide to WHO HAEM5

**DOI:** 10.1515/medgen-2024-2006

**Published:** 2024-03-06

**Authors:** Eugen Tausch, Cristina López, Stephan Stilgenbauer, Reiner Siebert

**Affiliations:** Ulm University Division of CLL, Department of Internal Medicine 3 Ulm Germany; Institut d’Investigacions Biomèdiques August Phi i Sunyer (IDIBAPS) Barcelona Spain; Ulm University, Division of CLL, Department of Internal Medicine 3 Ulm Germany; Ulm University and Ulm University Medical Center Institute of Human Genetics Ulm Germany

**Keywords:** WHO HAEM5, Chronic lymphocytic leukemia, Plasma cell neoplasms, Multiple Myeloma

## Abstract

The 5th edition of the World Health Organization Classification of Haematolymphoid Tumours (WHO-HAEM5) provides a revised classification of lymphoid malignancies including chronic lymphocytic leukemia (CLL) and plasma cell myeloma/multiple myeloma (PCM/MM). For both diseases the descriptions of precursor states such as monoclonal B-cell lymphocytosis and monoclonal gammopathy of uncertain significance (MGUS) have been updated including a better risk stratification model. New insights on mutational landscapes and branching evolutionary pattern were embedded as diagnostic and prognostic factors, accompanied by a revised structure for the chapter of plasma cell neoplasms. Thus, the WHO-HAEM5 leads to practical improvements of biological and clinical relevance for pathologists, clinicians, geneticists and scientists in the field of lymphoid malignancies. The present review gives an overview on the landscape of genetic alterations in CLL and plasma cell neoplasms with a focus on their impact on classification and treatment.

## Chronic lymphocytic leukemia

Chronic lymphocytic leukemia (CLL) is an indolent leukemic lymphoma and the most common leukemia in the western world. The World Health Organization (WHO) Classification of Haematolymphoid Tumours in its 5^th^ edition embedded the disease into the classification of lymphomas and therefore provides definitions for pathologists and clinicians [1]. Here CLL and its non-leukemic (i. e. nodal) presentation named small lymphocytic lymphoma (SLL) are within the category of mature B-cell neoplasms and together with monoclonal B-cell lymphocytosis (MBL). While CLL and SLL were once considered distinct entities, it is now widely accepted that they represent different manifestations of the same disease. The genetic and morphologic differences between CLL and SLL are minimal and treatment approaches for CLL and SLL are often considered together. Therefore the data and recommendations in this article are valid for both CLL and SLL, even if only CLL is mentioned in the text. While the characteristic immunophenotype of CLL and CLL-type MBL includes expression of CD19, CD20, CD5 and CD23, frequently accompanied by CD43, CD79b, CD81, CD200 and ROR1, the absolute B-cell count defines the specific subgroup [13, 24]: A count below 0.5 × 10^9 /L outlines low-count MBL, which comprises a clonal B-cell expansion commonly found in the elderly population. As for the majority of these cases neither a progression to CLL nor a need for therapy occurs, this entity should not be considered as a malignant disease state. This is different for CLL-type MBL defined by B-cell counts between 0.5x10^^9^ /L and 5x10^^9^ /L, which can be seen as a premalignant state with an annual risk for progression to CLL of about 1 % [11, 27]. MBL not characterized by the CLL phenotype can in some cases be classified as another lymphoma i. e. marginal zone lymphoma (MZL), while in other cases it remains an unspecified non-CLL-type MBL. While most people with MBL will probably never need treatment, the detection is still informative as it is associated with immune impairment including a poorer response to vaccination.

In contrast to MBL, CLL is defined by a clonal B-cell lymphocytosis of at least 5x10^9 /L and the course of disease is very heterogeneous. In WHO-HAEM5 CD5 positive cases of former B-prolymphocytic leukemia (B-PLL) defined by >15 % prolymphocytes in blood/bone marrow are now incorporated into the diagnosis of CLL, provided an appropriate phenotype and the exclusion of an *CCND1* rearrangement are demonstrated [1]. However, the large majority of cases of CLL have a mature phenotype and genetic markers are among the strongest prognostic factors to predict time to first treatment (TTFT), progression free survival (PFS) and overall survival (OS) [13]. Among different recurrent aberrations and somatic gene mutations, the mutation status for *TP53*, and/or deletion of 17p/*TP53* and the mutation status of the immunoglobulin heavy chain (IGH) locus variable region (IGHV) have been extensively tested for their prognostic and predictive significance and are recommended as standard diagnostics for CLL in WHO-HAEM5.

**Table 1:**
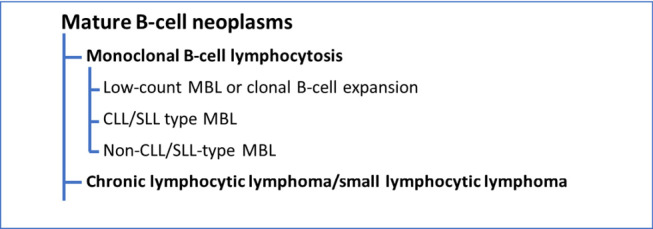
WHO Classification of MBL and CLL

## Hypermutation of clonally rearranged IGHV region is prognostic in CLL

**Figure 1:**
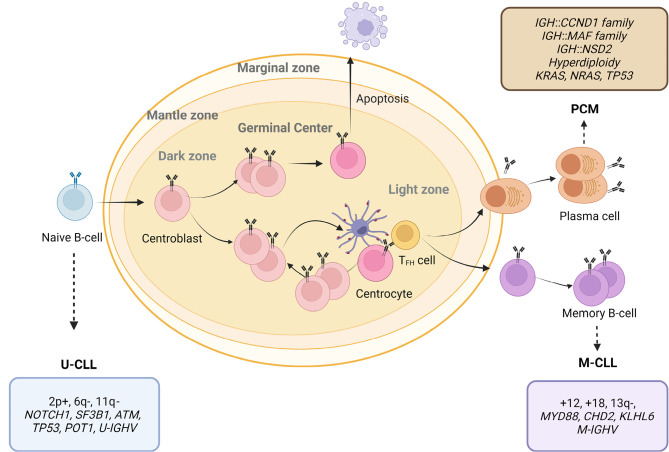
Chronic lymphocytic B-cell leukemia and plasma cell myeloma, cell of origin and main genetic aberrations. B-cell maturation progresses from the naïve B-cell to memory B-cells and plasma-cells. The most frequently altered genes or genomic regions are included in the box belonging to each entity or subtype. U, unmutated; M, mutated, CLL, chronic lymphocytic leukemia; PCM, plasma cell myeloma.

The B-cell receptor (BCR) is a central player in the development and progression of CLL. It is a protein complex, consisting of a membrane bound combination of immunoglobulin heavy and light chains, expressed on the surface of B cells that are normally responsible for antigen-specific immune response. The immunoglobulin heavy chain variable region (IGHV) encodes the variable region of the BCR. Mutations in the IGHV gene can lead to changes in the specificity and affinity of the BCR and are of strong clinical relevance due to their independent prognostic role [14]. Therefore, in a diagnostic laboratory setting, CLL DNA is extracted and IGHV amplified and sequenced with a set of consensus primers in accordance to the current recommendations of the European Research Initiative on CLL (ERIC) [26]. The sequence is compared to the closest related germline sequence. IGHV is considered as mutated, if at least 2 % of the IGHV nucleotides are different from those of the germline sequence corresponding to a homology of <98 %, while patients with fewer mutations have an unmutated IGHV status (homology >98 %) (**Figure 1**). Here the alignment to the correct germline sequence is the critical point, as comparison to a different sequence results in a different homology percentage and therefore sometimes to a different interpretation of the IGHV mutation status. The use of the IMGT/V-Quest tool (https://www.imgt.org/IMGTindex/IMGTHighV-QUEST.php) for this crucial step is strongly recommended, as it provides not only the correct sequence cross referenced with V-Base and GenBank for the alignment, but also calculates the percentage of homology. In addition to the recommended primer set, different labs have established their routine work flow with framework primers which are not covering the full transcribed region, but deliver comparable results in a majority but not all cases.

The IGHV mutation status is a powerful prognostic factor in CLL, as patients with mutated IGHV have a significantly better prognosis than patients with unmutated IGHV. This is shown for PFS and with longer follow up even for OS with time limited treatment regimens such as chemotherapy, venetoclax-based regiments and in part also for continuous BTK inhibitor treatments [28, 2, 15]. At diagnosis, around 50 % of patients have a mutated IGHV status; however, the status is not necessary for diagnostic purposes. As different national and international guidelines provide treatment recommendations according to the IGHV mutation status, the test result must be available at the time point of treatment initiation. IGHV mutation is considered as a stable parameter in the course of CLL and therefore a single analysis to categorize in IGHV-mutated or -unmutated is adequate.

In addition to IGHV, the light chain variable region (IGLV) is also a part of the BCR. CLL cells expressing IGLV3–21 account for up to 20 % of CLLs/SLLs and frequently have a mutation in the IGLV3-21 R110 locus. Patients with IGLV3-21R110 have short TTFT and OS indicating an unfavorable prognosis independent of IGHV mutational status. This subset of CLL is characterized by intermediate epigenetic subtype and specific driver alterations and is strongly associated with IGHV subset #2.

Despite the use of different VDJ rearrangements, B-cell receptors can have a homologous VH CDR3 region and thus be stereotyped. For approximately 30 % of CLL patients, such a stereotyped BCR can be identified and assigned to a subset. The best-characterized CLL stereotyped subsets include subset #1, #2, #4, and #8, which have been associated with distinctive clinical features, genetic factors, expression profiles and different outcomes [16]. Subset #2 represents the largest stereotyped subset, still only affecting less than 5 % of cases in most cohorts; it has been associated with an aggressive clinical course, irrespective of the IGHV hypermutation status. Subset #4 is the largest subset of mutated IGHV CLL and is characterized by lower CD38 expression and the absence of *NOTCH1*- and *SF3B1* mutations, while subset #8 has a higher risk for transformation into an aggressive lymphoma (Richter transformation) [16]. Overall, analyzing subsets in CLL can provide valuable information for improving risk stratification and understanding the disease’s heterogeneity. However, in the majority of cases the subset cannot be assigned. Furthermore, there is insufficient data on the prognostic impact with the currentlywidely used modern therapeutic agents includingthe BTK inhibitors ibrutinib, acalabrutinib, zanubrutinib or with the BCL2 inhibitor venetoclax.

## Recurrent chromosomal aberrations are routinely diagnosed via fluorescence in situ hybridization (FISH)

CLL is characterized by recurrent chromosomal aberrations, which affect >80 % of CLL cases when using “interphase cytogenetics” by FISH. FISH is the analysis routinely used to assess deletions in the long arm of chromosome 13 [del(13q)] and 11 [del(11q)], the short arm of 17 [del(17p)] as well as gain of chromosome 12, which are the most common genomic aberrations in CLL [10]. Although recurrent in CLL, they can also be found on other lymphomas and are therefore not pathognomonic. FISH is performed mainly on peripheral blood, however, bone marrow or lymph node aspirates can also provide a source for material in aleukemic phases of CLL or SLL.

At diagnosis cytogenetic FISH examination is not mandatory but can be carried out to confirm the diagnosis, especially if flow cytometry or cytomorphology are not sufficient to distinguish from other indolent lymphomas. Therefore, FISH analysis of the IGH locus on chromosome 14q32 is supportive. While translocations involving the IGH locus affect less than 5 % of cases of CLL, t(11;14)(q13;q32) leading to *IGH*::*CCND1* juxtaposition, in the setting of a small B-cell neoplasm, is diagnostic of mantle cell lymphoma, and t(14;18)(q32;q21) leading to *IGH*::*BCL2* juxtaposition is typical for leukaemic follicular lymphoma [1]. Whether other IGH-translocations determine distinct diseases sometimes associated with “atypical CLL”, like t(14;19)(q32;q13) leading to *IGH*::*BCL3* juxtaposition or del(14)(q24q32) associated with *IGH*::*ZFP36L1* fusion warrants further analyses.

Different national and international guidelines mandate FISH analysis at least for del(17p) for every patient before initiation and every next line of therapy (Hallek et al. 2018). Del(17p) affects the locus of the tumor suppressor gene *TP53* and is a negative prognostic factor under time-limited treatment including chemotherapy and venetoclax based regimen and partly also for continuous BTK inhibitors [10, 28, 29, 18]. Repetition before each new line of therapy is necessary, as patients can acquire del(17p) through clonal evolution during the course of the disease. Therefore, the incidence at CLL diagnosis is at 5 % while in heavily pretreated patient groups it affects about 50 % of patients.

Deletion of the long arm of chromosome 11, i. e. del(11q), is also a negative prognostic factor affecting about 20 % of patients at first treatment. The region contains the genes *ATM, NPAT, CUL5* which are discussed in the context of CLL pathogenesis. However, the supportive data derives from treatment with chemotherapy, while studies with BTK- and BCL2-inhibitors have mainly not shown any prognostic impact [28, 29, 7]. Further chromosomal changes are also analyzed routinely. This includes deletion in 13q with a minimal region of loss affecting the *DLEU2*-mir-15-16 gene cluster, which is found in heterozygous state in around half of all patients and in homozygous state with lower frequency. Del(13q) if appearing as a single aberration is associated with a very indolent course of disease. An intermediate risk is outlined for patients with trisomy of chromosome 12q and without any of these recurrent aberrations which is widely termed “normal karyotype” not considering aberrations in other loci (both 20 % incidence). In a regression model the presence of a favorable and unfavorable marker did not balance the prognosis, but the aberration with the most inferior prognosis specifies the outcome, therefore CLL with del(17p) has a worse prognosis than CLL with del(11q) or +12, followed prognostically by CLL with normal karyotype, and CLL with del(13q) with best prognosis (Döhner et al. 2000). This hierarchical model is widely used by diagnostic labs to estimate the outcome of CLL patients.

## Complex karyotype has prognostic relevance but is not specified in CLL/SLL

Other abnormalities such as del(6q), del(8p), del(9p), +18, +19 are recurrent with lower frequency and less well characterized in their biologic and clinical significance. However, also the total number of aberrations in the genome of a CLL patient is important. Notably, although WHO-HAEM5 describes the genomic complexity as a desirable additional investigation, it does not provide a definition for complex karyotype in CLL, in contrast to AML/MDS. This also applies in a similar way to many international and national guidelines with interpretation of (highly) complex karyotype as high risk CLL with consequences for the preferred therapy. In accordance with specifications for AML/MDS the presence of at least three chromosomal aberrations is widely defined as complex karyotype, five and more aberrations are termed highly complex. A shorter OS and shorter PFS after chemotherapy have been documented for cases with 5 or more aberrations [3, 12]. For 3 or 4 aberrations data is less clear, as in some trials with chemotherapy, complex karyotype is also associated with shorter PFS. However, there is still insufficient data on genomic complexity as a prognostic factor with novel compounds, especially when given as a continuous treatment [7, 12]. Furthermore, not all aberrations seem to play the same role. Chromosomal gains, especially of 12, 18 and 19, have a favorable prognosis and also for balanced translocations an adverse impact on outcome is not conclusive. Importantly, CLL with unmutated-IGHV have higher number of genomic alterations than those with mutated-IGHV, and harbor different genetic alterations. For example, gain of 2p and losses of 6q and 11q are more frequent in unmutated-CLL, in contrast to trisomies 18 and 19 more frequently observed in mutated-CLL (**Figure 1**). To assess these chromosomal aberrations different methods have been introduced including chromosome band analysis (CBA) after specific stimulation with CpG+IL2 or CD40L, genomic microarrays (CMA), whole genome sequencing (WGS) and optical genome mapping. While the latter two are not widely used in routine diagnostics yet, CBA and CMA have both different advantages [23]. CMA has a higher resolution to detect small gains and losses compared to CBA, but lacks the ability to detect balanced translocations. However, as both balanced translocations and minor aberrations not covered by CBA seem to be of insignificant prognostic value, comparative analysis showed a high concordance between both methods to categorize CLL into complex or highly complex karyotype.

## *TP53* mutation associated with shorter survival

The genomic landscape of CLL has been studied in detail in recent years and several publications have contributed to our understanding of the disease. Integration of genomic, transcriptomic and epigenomic data from 1148 patients identified more than 200 candidate genetic drivers of CLL [17]. Segregation into new IGHV subtypes with distinct genomic landscapes and leukemogenic trajectories and its association with outcome revealed insights into oncogenesis and prognostication. Further exploration of noncoding areas extended the catalog of cancer drivers including structural variants and global genome features associated with response, disease relapse and transformation [25]. Key signaling pathways for CLL have been identified on the basis of various gene mutations: In addition to BCR (*CARD11, NFKBIE*), TLR (*MYD88*) and MAPK (*BRAF, RAS*) receptor signaling, NOTCH (*NOTCH1, FBXW7*) and the non-canonical NF-kb signaling pathway (*BIRC3, TRAF3*) play an important role. Furthermore epigenetic modification (*CHD2, ARID1A*), transciptional regulation (*EGR2, MED12*) and RNA processing (*SF3B1, XPO1*), chromatin modification (*CHD2*) and ribosomal alterations (*RPS15*) are frequently affected. Finally, mutations in genes for cell cycle control and DNA repair (*TP53*, *ATM*) are prognostically important. Still a great deal of scientific work is required in order to use the complex findings in routine practice in a meaningful way, to improve patients’ outcome. Predictive markers guiding choice of therapy are yet rare and even with the most frequent mutations in CLL, affecting *SF3B1, NOTCH1* and *ATM,* the impact on clinical decisions is modest. This has different reasons: For some of the drivers there is strong association with unmuted (U-) IGHV (i. e. *BIRC3, NOTCH1*) while others are found with higher incidence in cases with specific aberrations: i. e. *ATM* mutations coincide with del(11q), *NOTCH1* mutations with +12 and mutated *TP53* with del(17p) cases. While significant in univariate analysis, only few markers are validated as independent prognostic factors in a multivariate testing [29, 28]. *TP53* alterations in particular are associated with shorter PFS and OS in studies with time limited venetoclax-based therapy and to a minor degree also with continuous BTK inhibitor therapy irrespective of other genetic risk factors. Also, in cases without del(17p), which accounts for about 50 % of *TP53* mutated CLLs, an adverse outcome is documented. Therefore it is not surprising, that in Cox regression models, mutated *TP53,* and frequently only del(17) and U-IGHV, remain of independent prognostic value [29, 28, 31]. Therefore these three factors and beta-2-microglobulin, age and clinical stage are implemented into the CLL-IPI, an international prognostic impact informative for TTFT, PFS and OS in CLL [15]. However, this was derived from data of the chemotherapy era and needs further validation in the context of novel compounds. Similarly the IPS-E including IGHV status, absolute lymphocyte count (ALC) and palpable lymph nodes characterizes the clinical course of early stage CLL [9].

## Clonal evolution can result in transformation to an aggressive lymphoma

Clonal evolution can affect gene mutations and chromosomal aberrations, but is not observed for the IGHV gene rearrangement. An increase in the incidence of *TP53* mutations is observed with an increasing number of therapy lines. Also, resistance mutations are observed under long-term treatment with kinase inhibitors. Such mutations in BTK typically affect the binding site [30, 4]. About 50 % to 80 % of patients under covalent inhibitors such as Ibrutinib develop a mutation in *BTK* after several years of therapy, mainly in the cysteine at position 481 [30]. While Ibrutinib, Acalabrutinib or Zanubrutinib show a lower affinity to C481S or C481F resulting in refractoriness, non-covalent inhibitors such as Pirtobrutinib are effective, but select for other mutations, usually of gatekeeper type, causing resistance (i. e. in protein position 528, 474, etc). Therefore, to inform a therapeutic sequence of covalent and non-covalent BTK-inhibitors, an analysis for resistance mutations can be informative. Hyperreactive mutations in *PLCG2* downstream of BTK lead to a constitutive pathway activation, but are rarely observed. Also resistance mutations in *BCL2* are observed with continuous venetoclax therapy. However, as BCL2 inhibitors are nowadays mainly used as a time limited therapy in combination with CD20 antibodies and resistance mutations are not observed in relapsed patients but only in CLL refractory to continuous treatment, a diagnostic testing for such variants is only justified in special situation.

The most concerning type of evolution results in a transformation into an aggressive lymphoma, typically diffuse large B-cell lymphoma, rarely Hodgkin lymphoma. These cases of so called Richter transformations share genetic markers of CLL (*TP53, NOTCH1, SF3B1*) with aberrations typically found in aggressive lymphoma (amp(1q23), amp(8q24), del(6q)) [21]. A vast majority of Richter cases are clonally related to the CLL in the same individual confirmed by the same IGHV rearrangement in CLL and the aggressive lymphoma or shared gene mutations. However, in few cases of confirmed CLL, the genetic and epigenetic profile of the subsequent DLBCL fits rather to de novo DLBCL. Similar to de novo DLBCL or Hodgkin lymphomas in patients without CLL, these clonally unrelated Richter cases have a high chance of cure with conventional chemoimmunotherapy, whereas Richter transformations clonally related to CLL respond poorly to chemotherapy and are usually fatal, especially in patients ineligible for allogeneic stem cell transplantation [6]. Therefore the occurrence of Richter transformation is the biggest challenge in the context of CLL and new treatment concepts for this disease are urgently required.

**Table 2:** Genetic alterations, method of detection and purpose/clinical relevance in CLL.

**Required Genetic Diagnostics**			
Type of diagnostics	Specific Test	Method	Purpose
Molecular Diagnostics	*TP53* mutation	NGS/Sanger	Prognostic marker; predictive i. e. vs. chemotherapy
	IGHV mutation status	GeneScan/Sanger + IMGT/V-Quest	Prognostic marker; predictive i. e. vs. chemotherapy
	IGHV subset analysis	Sanger + ARResT	Prognostic marker
Cytogenetics	del(17p)	FISH	Prognostic marker, predictive (vs. chemotherapy)?
	t(14;18), t(11;14)	FISH	Characteristic of follicular lymphoma and mantle cell lymphoma
	
**Useful diagnostics**			
Type of diagnostics	Specific Test	Method	Purpose
Cytogenetics	Genomic complexity/Complex karyotype	CMA/Chromosome banding	Prognostic marker
Molecular Diagnostics	Mutations in *SF3B1, NOTCH1, ATM, BIRC3, RPS15, EGR2, NFKBIE*	NGS	Prognostic marker
	*BTK, PLCG2, BCL2* mutations	Sanger/NGS	Compound-specific resistance mechanisms

t, translocation; del, deletion; NGS, next generation sequencing; Sanger, sanger sequencing; CMA, genomic microarrays

## Plasma cell neoplasms

In contrast to CLL, plasma cell neoplasms derive from terminally differentiated B-cells characterized by production of monoclonal immunoglobulin, typically IgG or IgA. This includes the full diagnosis of plasma cell myeloma/multiple myeloma (PCM/MM), but also precursors such as smoldering myeloma, monoclonal gammopathy of undetermined significance (MGUS), of renal significance (MGRS) and plasma cell related disorders such as cold agglutinin disease (CAD), amyloidosis and paraneoplastic syndromes (**Table 3**). In WHO-HAEM5 the structure of the chapter plasma cell neoplasms and other diseases with paraproteins was revised. This included a restructuring of all myeloma-related disorders as listed above and addition of a subdivision of MGUS into subgroups defined by genetic abnormalities. CAD, IgM MGUS and Non-IgM MGUS are subsumed as monoclonal gammopathies while diseases with monoclonal immunoglobulin deposition include immunoglobulin-related (AL) amyloidosis and monoclonal immunoglobulin deposition disease previously known as light/heavy chain deposition disease. Alpha, Gamma and Mu heavy chain diseases remain a distinct subgroup separated from plasma cell neoplasms, which in addition to plasmacytoma and plasma cell myeloma now includes three different paraneoplastic syndromes: POEMS (Polyneuropathy, Organomegaly, Endocrinopathy, M-protein, and Skin changes), TEMPI (telangiectasias, elevated erythropoietin level and erythrocytosis, monoclonal gammopathy, perinephric fluid collections, and intrapulmonary shunting) and AESOP (Adenopathy and Extensive Skin Patch Overlying a Plasmacytoma).

**Table 3:**
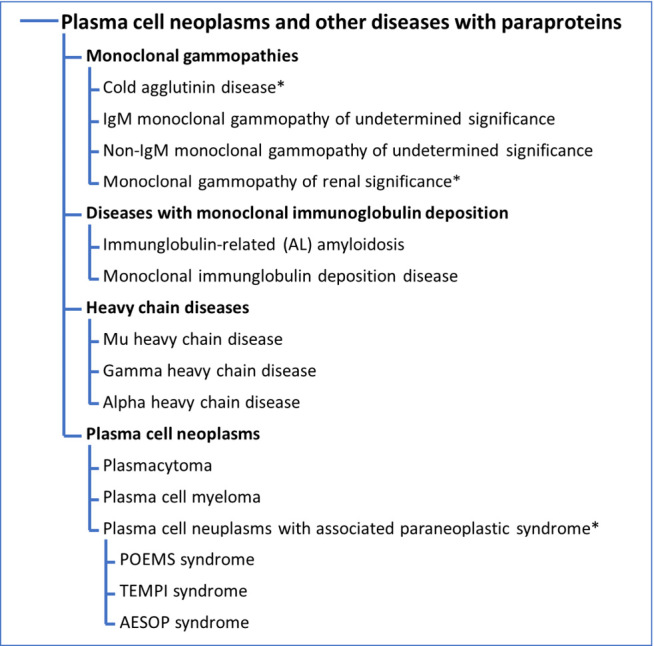
WHO Classification of Plasma cell neoplasms and other diseases with paraproteins. Diagnosis with * were not previously included in the revised 4^th^ edition of WHO Classification. Please note that FISH should be performed on CD138 positive selected cells.

## MGUS is reclassified depending on the genetic background

MGUS is still defined as a paraprotein <30g/L and without bone marrow infiltration of at least 10 % by plasma cells. Risk parameters for progression to myeloma are identified in 1) an abnormal serum free light chain ratio, 2) IgA or IgM type MGUS and 3) a serum M-protein >1.5g/dl. While the risk of progression to symptomatic disease stage without these risk factors is below 5 % in 20 years the chance is 50–60 % with presence of all three factors [22].

CAD was defined as a new entity in WHO-HAEM5 characterized by an indolent lymphoproliferative disorder in bone marrow with a specific cold agglutinin IgM antibody with IGHV 4–34 isotype. Primary cold agglutinin disease lacks the presence of splenomegaly, markedly enlarged lymph nodes and *MYD88^L265P^* mutation, which all point to the presence of a lymphoma and therefore a secondary CAD. In contrast *KMT2D* and *CARD11* mutations are frequently detected in primary CAD in addition to trisomies of chromosomes 3, 12, and 18 [19].

## Genetic lesions in MGUS and plasma cell / multiple myeloma

Plasma cell myeloma (PCM), clinically mostly designated multiple myeloma (MM), is consistently preceded by clinically recognized precursor lesions: monoclonal gammopathy of undetermined significance (MGUS) and/or the asymptomatic disease stage smoldering MM (SMM) (**Figure 1**). In contrast to MGUS or MGRS, SMM is characterized by significant (>10 %;<60 %) bone marrow infiltration but lack a treatment indication defined by the SLiM-CRAB criteria. While the annual risk of progression from SMM to PCM amounts to 10 %, about one third of patients with SMM have not progressed in the first 10 years after diagnosis and remain in an inactive disease stage. However, the majority develops hypercalcemia, renal impairment, anemia and/or bone lesions or prominent laboratory values associated with need for therapy. Such indication includes bone marrow infiltration of plasma cells >60 %, a light chain ratio >100 or more than one focal lesion detected by MRI. However, the genetic background of SMM and MMis close to identical: The most frequent chromosomal translocations involve the *IGH* locus, and the partners identified are the i) CCND family, including t(11;14)/*CCND1*::*IGH*, t(12;14)/*CCND2*::*IGH*, t(6;14)/*CCND3::IGH* and light chain variants*;* ii) MAF family, corresponding to t(14;16)/*IGH*::*MAF*, t(8;14)/*MAFA*::*IGH*, t(14;20)/*IGH*::*MAFB* and light chain variants*;* iii)* NSD2* rearrangement, t(4;14)[24]. The described chromosomal translocations are usually detected using FISH on CD138 positive selected cells or FICTION (Fluorescent immunophenotyping and interphase cytogenetics) in assays on CD138 positive selected cells. PCM/MM lacking an *IGH* rearrangement are frequently characterized by hyperdiploidy (trisomies of chromosomes 3, 5, 7, 9, 11, 15, 19 and 21). *IGH* rearrangements, hyperdiploidy and chromothripsis, therefore, are primary genetic alterations while monosomies of chromosomes 13, 17, 14, and focal deletions oncogenes such as of 17p (*TP53*), 17q and 1p, and gains or amplifications of chromosome 1q are described as late events conferring further selective advantage [5, 8]. While early events are frequently also observed in MGUS, late events have a higher incidence in symptomatic PCM as a result of clonal selection over time. Monosomy 13 or deletion 13q14 is identified in a high proportion of the cases (50 %) and can be an early event but also a secondary alteration. An *IG*::*MYC* rearrangement is also highly prevalent.

The mutational landscape of PCM/MM is very heterogeneous. Overall 87 % of all MM had at least one of 61 drivers identified in a comprehensive analysis of driver events with an average of 2 driver mutations per patient. A majority of these driver point mutations were found to be present in subclones suggesting their involvement in later stages of cancer development. Frequently altered genes are *NRAS, KRAS, TP53, DIS3, FAM46C,* and* BRAF* [20]*.* For SMM mutations in the MAPK pathway (*NRAS*, *KRAS*), DNA repair pathway (*TP53*, *ATM*) as well as MYC aberrations (amplification, translocation, *FUBP1* mutations) associated with early progression. Similar to CLL and other B-cell malignancies recurrent mutations also cluster in NF-kB pathway, protein processing and cell cycle control. A distinctive pattern of missense mutations clustered in linker histones may have a disruptive effect on regulation of chromatin structure [8]. With t(4;14), del(1p), del(14q), del(16p), del(17p) and biallelic deletions of *TP53, RB1, CDKN2A* etc. further drivers to transition to PCM were identified.

Finally, the presence of high-risk markers results in a clinical decisions such as more aggressive treatment regimens including a tandem autologous transplantation up front. Therefore, at a minimum, del(17p), t(4;14)*IGH*::*NSD2* and t(14;16)*IGH*::*MAF* must be covered by FISH analysis to perform risk stratification. Normally testing for t(14;20) and amplification of 1q21 is also incorporated. However, as alluded to above, recent data has introduced different factors for application in routine diagnostics following a separate, favorable prospective analysis.

## Conclusions

A series of comprehensive genomic studies in CLL and plasma cell neoplasias has recently described the landscape of these alterations. Nevertheless, only a small subset of these changes have diagnostic, prognostic or therapeutic importance. Up-to-date diagnostics includes detection of these changes mostly by a combination of FISH- and (targeted) sequencing based approaches.

**Table 4:** Clinical relevance and detection method of genetic alterations in plasma cell neoplasms and other disease with paraproteins. Of note, molecular studies using next generation sequencing (NGS), sanger sequencing, fluorescent in situ hybridization, and karyotype, should be performed in sorted CD138 cells in plasma cell neoplasms

**Entities**		**Genetic alterations**	**Clinical relevance**	**Method**
**Monoclonal gammopathies**				
**Cold agglutinin disease (CAD)**		No *MYD88* (p.L265) mutation	Useful for diagnosis and differential diagnosis with other B-cell neoplasms	NGS/Sanger
*KMT2D* and *CARD11* mutations	Appropriate for diagnosis			
Trisomies 3, 12, 18	Useful for diagnosis	FISH/Karyotype
**IgM monoclonal gammopathy of undetermined significance (MGUS)**		t(11;14)/*CCND1*::*IGH* translocation	Appropriate for diagnosis	FISH
No *MYD88* (p.L265) mutation	Useful for diagnosis and differential diagnosis with other B-call neoplasms	NGS/Sanger
*CXCR4* mutation	Appropriate for diagnosis and differential diagnosis with CAD	
**Disease with monoclonal immunoglobulin deposition**				
	**Immunoglobulin-related (AL) amyloidosis**	t(11;14)/* CCND1*::*IGH* translocation	Useful for diagnosis Higher frequency compared to other PC neoplasms	FISH
**Heavy chain disease**				
**Gamma heavy chain disease**		Complex karyotype	Useful for diagnosis	Karyotype
No *MYD88* (p.L265) mutation	Useful for diagnosis and differential diagnosis with lymphoplasmacytic lymphoma	NGS/Sanger
**Plasma cell neoplasms**				
**Plasma cell myeloma**		*IGH*::*CCND* family translocations	Useful for diagnosis and associated with standard prognosis	FISH
*IGH*::*MAF* family translocations	Useful for diagnosis and associated with poor prognosis			
*IGH*::*NSD2* translocation	Useful for diagnosis and associated with poor prognosis			
Hyperdiploid (Trisomies 3, 5, 7, 9, 11, 15, 19, 21)	Useful for diagnosis and associated with favorable prognosis	FISH/Karyotype
del17p/* TP53* mutations Gain 1q	Associated with progression and relapse	FISH/NGS

t, translocation; NGS, next generation sequencing; Sanger, sanger sequencing; PC, plasma cell
